# Exercise in Patients with Subclinical Atherosclerosis: Mechanisms, Clinical Evidence, and Practical Recommendations

**DOI:** 10.1007/s11883-026-01464-6

**Published:** 2026-09-08

**Authors:** Thaddeus Gagaring, Olivia Drummond, Gaetano Santulli

**Affiliations:** School of Medicine, City University of New York, Manhattan, NY 10031 USA

**Keywords:** Aging, Carotid plaque, Coronary artery calcium, Endothelial dysfunction, Exercise, Inflammation

## Abstract

**Purpose of Review:**

Subclinical atherosclerosis represents an important stage in the continuum of cardiovascular disease, characterized by structural or functional vascular abnormalities in the absence of overt clinical events. This review examines the biological mechanisms and clinical evidence supporting exercise as both a preventive and potential disease-modifying intervention in individuals with imaging-detected subclinical atherosclerosis, with particular emphasis on endothelial function, vascular remodeling, inflammation, plaque composition, and cardiovascular risk.

**Recent Findings:**

Increasing evidence indicates that exercise exerts vascular effects that extend beyond conventional risk-factor modification. Exercise improves endothelial function, vascular compliance, and nitric oxide bioavailability through shear stress-mediated activation of endothelial nitric oxide synthase (eNOS), while also modulating oxidative stress, myokine signaling, vascular inflammation, and macrophage phenotype. These effects may promote plaque stabilization and influence atherosclerotic lesion composition. Importantly, exercise-associated increases in coronary artery calcium observed in highly active individuals may reflect a shift toward more densely calcified and potentially more stable plaque rather than an accumulation of high-risk lipid-rich lesions. Clinical and meta-analytic evidence further suggests modality-specific effects, with interval training showing favorable effects on arterial stiffness and combined aerobic-resistance training improving endothelial function. Exercise may therefore complement pharmacological risk-factor modification by targeting vascular pathways that are not fully addressed by conventional therapies.

**Summary:**

Exercise should be considered an integral component of cardiovascular risk management in individuals with subclinical atherosclerosis rather than solely a primary-prevention strategy. Its potential benefits extend from improving vascular function and reducing inflammation to modifying plaque characteristics and promoting plaque stability. Future studies should determine whether exercise prescriptions can be individualized according to atherosclerotic burden, plaque phenotype, imaging characteristics, and circulating biomarkers to establish the optimal exercise modality, intensity, and dose across different patient populations.

**Graphical Abstract:**

**Figure:**
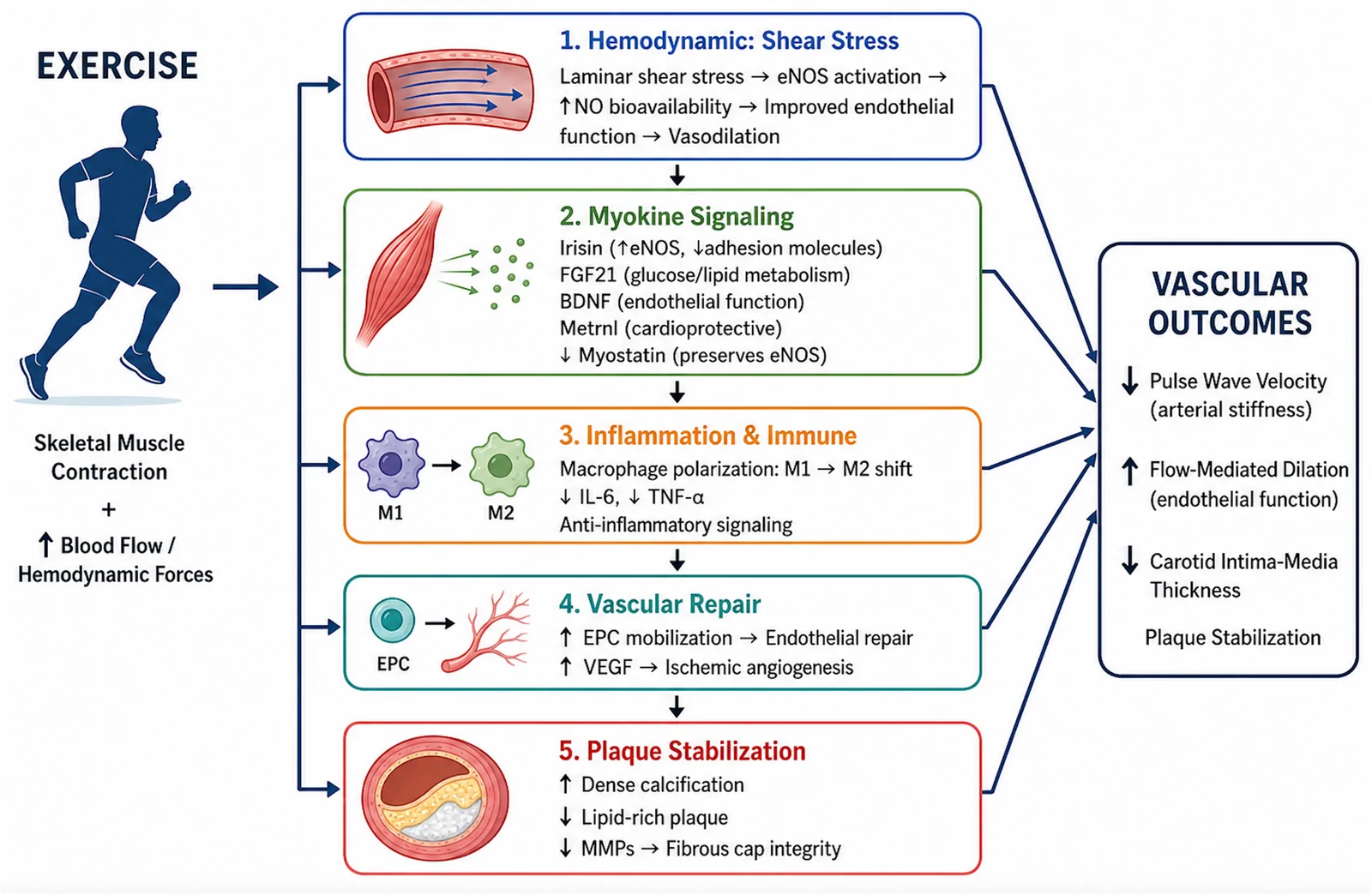

## Introduction

Atherosclerotic cardiovascular disease (ASCVD) remains the leading cause of mortality and disability worldwide despite major advances in prevention and treatment. Among its most important modifiable risk factors, low-density lipoprotein cholesterol (LDL-C) contributes substantially to the global burden of cardiovascular disease [1]. The persistent burden of ASCVD underscores the importance of identifying vascular disease before individuals present with myocardial infarction or stroke. Atherosclerosis is a progressive process that may begin years before the onset of clinical symptoms, with traditional cardiovascular risk factors contributing to structural and functional changes within the vasculature. For example, sustained elevations in LDL-C and systolic blood pressure can promote the development of detectable plaque and the progression of subclinical atherosclerosis [2]. This transition is clinically important because individuals may harbor a substantial atherosclerotic burden while remaining asymptomatic and unaware of their underlying disease. Atherosclerosis without clinical manifestations is generally termed subclinical atherosclerosis and may serve as an early marker of atherosclerotic burden. The principal distinction between subclinical and clinical atherosclerosis is the presence of clinically manifest ischemic disease, such as myocardial infarction, stroke, or chronic limb-threatening ischemia [3].

Early detection of subclinical atherosclerosis creates an opportunity for timely intervention. Exercise has traditionally been emphasized as a strategy for primary prevention, particularly among individuals at elevated cardiovascular risk who have not yet developed overt disease. Increasing evidence, however, suggests that exercise may also influence disease progression after subclinical atherosclerosis has been identified [4]. This review therefore examines exercise not only as a primary prevention strategy but also as a potential active, disease-modifying intervention for individuals with established subclinical atherosclerosis.

We searched PubMed/MEDLINE, Scopus, Web of Science, and the Cochrane Library from database inception through August 2026 using combinations of the following search terms: “exercise,” “exercise training,” “physical activity,” “aerobic exercise,” “resistance training,” “high-intensity interval training,” “subclinical atherosclerosis,” “endothelial function,” “endothelial dysfunction,” “coronary artery calcium,” “carotid intima-media thickness,” “pulse wave velocity,” “flow-mediated dilation,” “arterial stiffness,” “plaque stability,” “plaque composition,” “myokines,” “irisin,” “FGF21,” “shear stress,” “macrophage polarization,” and “endothelial progenitor cells.” Additional articles were identified by reviewing the reference lists of retrieved papers and key guidelines.

## What is Subclinical Atherosclerosis?

Subclinical atherosclerosis refers to the accumulation of atherosclerotic plaque and related vascular abnormalities before the development of overt clinical manifestations [3]. These abnormalities include structural features, such as atherosclerotic plaque, arterial wall remodeling, and vascular calcification, as well as functional alterations, including endothelial dysfunction and arterial stiffness [5]. Preventing progression of subclinical ASCVD requires accurate disease characterization, effective intervention, and longitudinal monitoring of individuals at risk. Several noninvasive techniques can identify subclinical disease, including coronary artery calcium (CAC) scoring and coronary computed tomography angiography (CCTA) [5], which can detect coronary atherosclerosis before the occurrence of cardiovascular events (Table 1). CAC scoring is one of the most widely used methods for detecting and quantifying coronary calcification and provides a standardized approach to calcium assessment on computed tomography [6]. The Agatston score is commonly used in asymptomatic adults, particularly those with borderline or intermediate cardiovascular risk, to quantify coronary calcium. However, CAC scoring has an important limitation: it does not detect noncalcified plaque. CCTA provides more comprehensive anatomical information and can identify nonobstructive, noncalcified plaque that is associated with cardiovascular risk [5, 7].

**Table 1:** Main modalities for the assessment of subclinical atherosclerosis

Modality	What it measures	Risk categories / thresholds	Advantages	Limitations	Refs.
CAC (Agatston score)	Quantifies calcified coronary atherosclerotic plaque using non-contrast CT; calcification is defined using a threshold of ≥ 130 HU and a lesion area > 1 mm².	CAC-DRS 0: very low risk; 1–99: mild risk; 100–299: moderate risk; ≥300: high risk. CAC scoring can guide preventive treatment in individuals with borderline or intermediate 10-year ASCVD risk.	Standardized, widely validated, and useful for guiding preventive treatment and risk reclassification.	CAC = 0 does not exclude the presence of non-calcified atherosclerotic plaque.	[5, 7]
CCTA	Characterizes coronary atherosclerotic plaque as calcified, non-calcified, or mixed and evaluates the presence and extent of coronary disease.	In one population-based study, 46% of asymptomatic adults ≥ 40 years had subclinical coronary atherosclerosis, including 36% with non-obstructive and 10% with obstructive disease.	Particularly effective for detecting non-calcified plaque, including in younger individuals and women; can reclassify patients from low/intermediate to higher cardiovascular risk.	Greater radiation exposure and higher cost than some other assessment modalities.	[5, 7]
Carotid ultrasound	Assesses carotid intima-media thickness (cIMT) and carotid plaque burden.	cIMT > 1 mm is associated with increased vascular risk. Globally, approximately 27.6% of adults have increased cIMT and 21.1% have carotid plaque.	Radiation-free, non-invasive, repeatable, widely available, and relatively inexpensive.	Operator-dependent and primarily assesses structural abnormalities; it does not directly capture functional endothelial dysfunction.	[8–10]
PWV	Assesses arterial stiffness, typically using carotid-femoral pulse-wave velocity measurements.	Used to stratify individuals into low-, intermediate-, and high-risk categories according to PWV values and clinical context.	Simple, non-invasive, reproducible, and provides an established measure of vascular stiffness.	Measurement and repeatability can vary according to the technique and device used.	[9, 10]
FMD	Measures flow-mediated dilation of the brachial artery as a functional assessment of endothelial function.	In one metabolic-risk comparison, FMD was 4.87% in metabolically unhealthy individuals versus 7.32% in metabolically healthy individuals, demonstrating its ability to distinguish vascular phenotypes.	Detects early functional endothelial dysfunction, potentially before overt structural vascular abnormalities become apparent.	Highly operator-dependent and technically demanding, with substantial methodological variability.	[11, 12]

Other imaging and physiological techniques can assess subclinical disease beyond the coronary arteries. Carotid ultrasonography is useful for cardiovascular and cerebrovascular risk stratification because it is noninvasive and can characterize carotid intima-media thickness (cIMT) and plaque burden [8]. Vascular function can also be assessed using flow-mediated dilation (FMD), a marker of endothelial function, and pulse wave velocity (PWV), a measure of arterial stiffness [9, 10]. Together, these approaches demonstrate that subclinical atherosclerosis encompasses both structural and functional abnormalities of the vasculature.

## Epidemiology

Subclinical atherosclerosis is common worldwide and can be detected in individuals who have not yet experienced clinical cardiovascular events. A global analysis estimated that carotid intima-media thickening affects approximately 27.6% of adults aged 30–79 years, whereas carotid plaque affects approximately 21.1% of the global adult population [13]. The prevalence and burden of subclinical atherosclerosis increase with age, while adverse cardiovascular health trajectories beginning during adolescence may contribute to greater atherosclerotic burden later in life [14]. Sex differences are also evident: men generally develop a greater plaque burden earlier in adulthood, whereas plaque progression in women accelerates after menopause [15]. Ethnic differences have likewise been reported, with higher subclinical plaque prevalence among non-Hispanic Black adults than among Hispanic adults and differences in CAC density across racial and ethnic groups [16, 17]. In addition, insulin resistance, chronic kidney disease, and chronic inflammation may accelerate atherosclerotic progression [18–20].

## Biological Mechanisms

### Pathophysiology of ASCVD

Subclinical atherosclerosis is initiated in part by endothelial dysfunction, a potentially reversible disruption of vascular endothelial homeostasis characterized by impaired regulation of vascular tone [21]. Under physiological conditions, endothelial cells regulate vascular tone in part through nitric oxide (NO), produced by endothelial nitric oxide synthase (eNOS). Exposure to cardiovascular risk factors promotes endothelial activation and reduces NO bioavailability, accompanied by increased expression of adhesion and chemotactic molecules [22], including intercellular adhesion molecule-1, monocyte chemoattractant protein-1, and vascular cell adhesion molecule-1 (ICAM-1, MCP-1, and VCAM-1).

Oxidative stress is a major driver of endothelial dysfunction. An imbalance between reactive oxygen species (ROS) and antioxidant defenses promotes eNOS uncoupling, further reducing NO bioavailability and impairing vasodilation while increasing vascular stiffness [22]. This oxidative environment promotes a proinflammatory endothelial phenotype through activation of pathways such as nuclear factor kappa B (NF-κB). Oxidized LDL (ox-LDL) can function as a damage-associated molecular signal and activate innate immune pathways involving cluster of differentiation 36, lectin-like oxidized LDL receptor 1, and the NLR family pyrin domain containing 3 inflammasome (CD36, LOX-1, and NLRP3) [23, 24]. These processes reinforce vascular inflammation and promote plaque progression.

The inflammatory cascade recruits circulating monocytes into the subendothelial space, where they differentiate into macrophages and internalize ox-LDL, generating lipid-laden foam cells that represent one of the earliest histologically recognizable features of atherosclerosis [25]. Vascular smooth muscle cells (VSMCs) can also undergo phenotypic switching within the intima and acquire a foam-cell phenotype [26]. These cells can release matrix metalloproteinases (MMPs), which may contribute to fibrous-cap degradation and plaque destabilization [27]. As lesions mature, extracellular vesicles released by smooth muscle cells and macrophages can promote the nucleation of microcalcifications, which are particularly abundant in vulnerable plaques [21]. Conversely, plaque stabilization and regression remain possible and may reduce plaque burden and markers of plaque vulnerability through mechanisms including enhanced cholesterol efflux and regulatory T-cell activity [28, 29].

## Biological Effects of Exercise on ASCVD

Exercise exerts several atheroprotective effects, with hemodynamic shear stress representing a central mechanism. Repeated increases in blood flow generate laminar shear stress (LSS) at the endothelial surface, promoting eNOS and sirtuin-1 signaling and supporting endothelial homeostasis and mitochondrial biogenesis [30–32]. These effects are consistent with evidence from a meta-analysis showing that aerobic exercise training can reduce oxidative stress and improve mitochondrial oxidative phosphorylation [33]. Exercise-induced shear stress can also suppress endothelin-1 expression, thereby favoring vasodilatory signaling [34]. Because eNOS is a major source of endothelial NO, shear stress-mediated activation contributes to improved endothelial function through increased NO bioavailability.

Beyond hemodynamic signaling, contracting skeletal muscle releases exercise-induced signaling molecules known as myokines. Irisin, one of the most extensively studied myokines, may improve endothelial function by increasing eNOS expression while reducing the expression of adhesion molecules and proinflammatory cytokines [35], including interleukin-6 (IL-6) and tumor necrosis factor-alpha (TNF-α). Other potentially atheroprotective myokines include fibroblast growth factor 21 (FGF21) and brain-derived neurotrophic factor (BDNF). FGF21 has important roles in glucose and lipid metabolism and may exert anti-atherosclerotic effects while contributing to exercise-induced cardioprotection [36, 37]. BDNF has been associated with improved endothelial function and lower cardiovascular risk and may additionally support neuronal survival, suggesting potential benefits of exercise for both cardiovascular and neurovascular health [38]. In contrast, myostatin (MSTN) may promote endothelial dysfunction by activating transforming growth factor-β signaling and inhibiting eNOS phosphorylation, thereby reducing NO production [37, 39]. Long-term exercise training can downregulate circulating myostatin and may therefore help preserve endothelial function. Exercise-induced adipokines, including apelin and meteorin-like protein (Metrnl), may provide additional vascular benefits. Apelin, particularly apelin-13, can improve lipid metabolism and exert anti-inflammatory effects by suppressing IL-6, TNF-α, and interleukin-1β, while Metrnl has also been associated with cardiovascular protective effects [37, 40–43].

In addition to improving endothelial function and reducing vascular inflammation and oxidative stress, exercise may promote vascular repair through ischemia-related angiogenesis, endothelial progenitor cell (EPC) mobilization, and macrophage polarization. Vascular endothelial growth factor (VEGF) is a key regulator of ischemic angiogenesis, promoting endothelial-cell migration and modulating vascular permeability [44, 45]. This process should be distinguished from intraplaque angiogenesis, which may contribute to plaque instability; ischemic angiogenesis instead supports the formation of new vascular networks in ischemic tissue [45]. VEGF signaling can also promote EPC mobilization, enabling these cells to participate in endothelial repair and maturation [46]. Exercise may initially increase macrophage polarization toward a proinflammatory M1 phenotype, whereas sustained training may favor a transition toward the reparative M2 phenotype [47]. This shift may promote anti-inflammatory signaling, tissue repair, and vascular homeostasis.

## Exercise and Subclinical ASCVD: Clinical Evidence

**Fig. 1:**
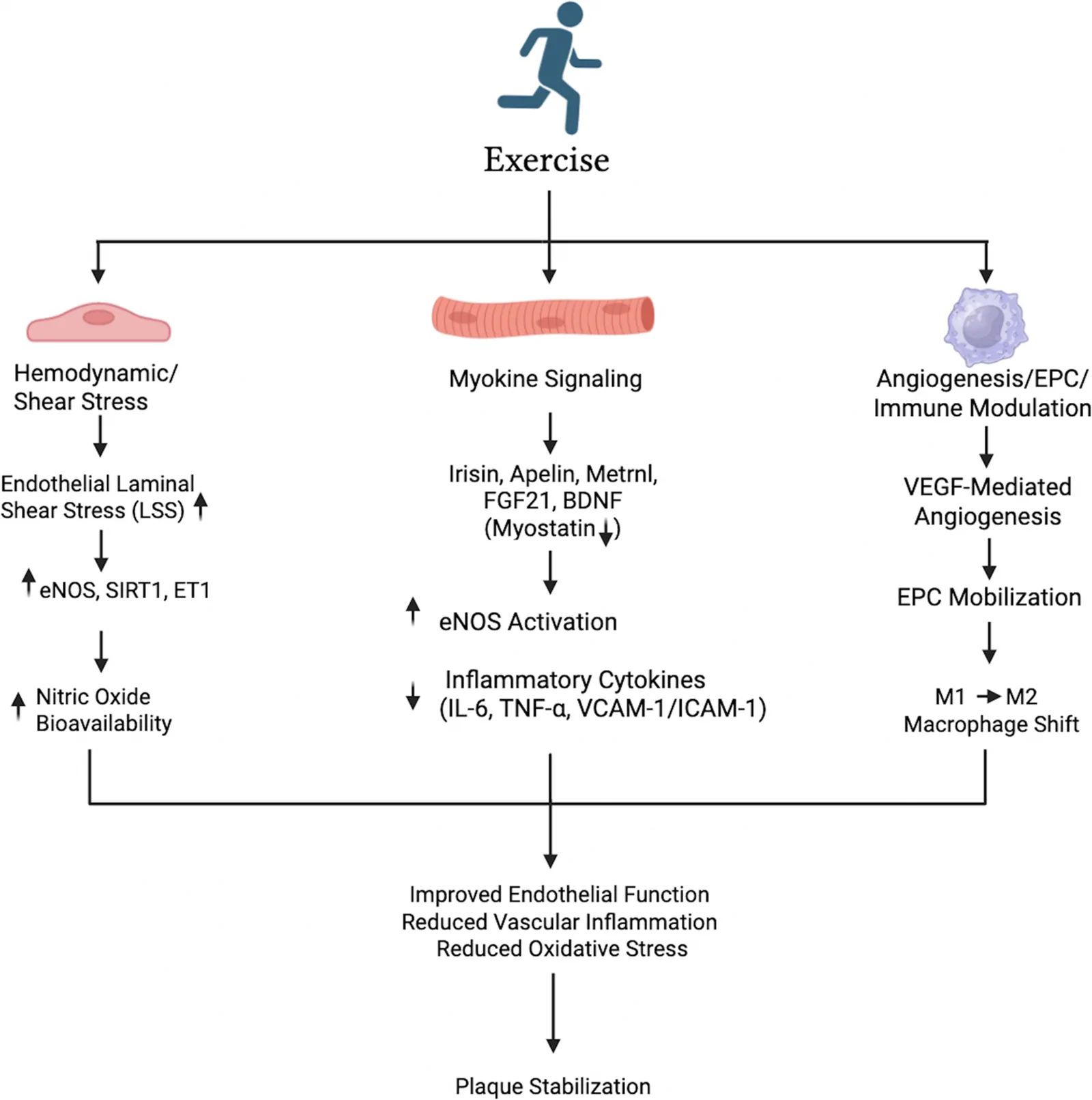
Main effects of exercise on the main pathophysiological aspects underlying subclinical atherosclerosis

While the mechanisms described above are well established at the cellular level (Fig. 1), a growing body of clinical evidence supports their translation into measurable vascular outcomes. The ATTICA study, which examined different patterns of physical activity in relation to long-term cardiovascular outcomes, found that aerobic health-enhancing physical activity was associated with a lower incidence of cardiovascular disease over 20 years of follow-up [48]. These findings support exercise as a cardioprotective intervention relevant to atherosclerotic disease. Exercise training was shown to improve FMD in adolescents, reduce PWV across age groups, and improve cIMT among adults younger than 60 years [49]. Collectively, these findings support the concept that exercise may function as an active, disease-modifying intervention rather than solely as a primary prevention strategy.

The relationship between exercise and CAC is more complex. Studies of endurance athletes have reported a relatively high prevalence of CAC despite otherwise favorable cardiovascular risk profiles (“calcium paradox”): masters athletes engaging in high volumes of endurance exercise, including long-distance running and cycling, may have higher CAC scores on computed tomography [50, 51]. Although the presence of CAC indicates subclinical atherosclerosis and is associated with cardiovascular risk, athletes may exhibit a greater proportion of densely calcified and potentially more stable plaque than sedentary individuals, who may have a larger burden of mixed or lipid-rich plaque [50, 52]. Nevertheless, findings from the *Cooper Center longitudinal study* [53] indicate that CAC remains associated with increased cardiovascular risk even among individuals with high cardiorespiratory fitness.

Beyond CAC, exercise has clinically measurable effects on other markers of subclinical ASCVD, including cIMT, PWV, and FMD. Specifically, exercise training lasting more than 6 months has been shown to reduce cIMT [54], high-intensity interval training performed two to three times per week can improve PWV [55, 56], and exercise training can improve FMD, with potentially greater effects from high-intensity aerobic exercise [57, 58]. These improvements may reflect reductions in age-related vascular inflammation, adverse lipid profiles, and oxidative stress [54, 57]. The clinical effects of exercise also extend to systemic inflammation and plaque composition. Indeed, exercise interventions have been shown to significantly reduce IL-6 and TNF-α in patients with heart failure [59]. Although these studies focused on heart failure, both cytokines are involved in atherosclerotic pathophysiology, providing biological support for an anti-inflammatory effect of exercise. Findings from the CENIT study and related analyses have further suggested an inverse association between fitness and lipid content within coronary atherosclerotic plaque, potentially indicating more favorable plaque composition [60, 61].

## Clinical Evidence by Modality and Intervention

### Different Exercise Modalities

Exercise is a central component of ASCVD prevention, and different forms of physical activity can improve vascular function, arterial stiffness, and cardiovascular risk factors [62, 63]. Moderate-to-vigorous aerobic exercise improves cardiovascular fitness and has been associated with meaningful improvements in endothelial function among middle-aged and older adults [64]. High-intensity interval training (HIIT), which alternates periods of vigorous exercise with recovery, can also improve vascular function in patients with established cardiovascular disease [65, 66]. However, HIIT should not be considered universally appropriate; exercise intensity should be matched to an individual’s cardiovascular status, functional capacity, and exercise tolerance [63, 67].

Resistance training is another important component of ASCVD prevention because it improves muscular strength and may contribute to overall vascular health. Combining aerobic and resistance exercise may provide broader benefits than either modality alone [68]. Different exercise strategies can produce distinct effects on vascular biomarkers: combined training may maximize improvements in endothelial function, whereas resistance and continuous endurance training may reduce cIMT [69–71]. In adults with type 2 diabetes, combining aerobic and machine-assisted resistance training can improve both vascular function and HbA1c [72, 73].

Lower-impact activities can also provide clinically meaningful cardiovascular benefits. Aquatic exercise can improve endothelial function and systemic and peripheral arterial stiffness, making it a potentially useful option for individuals who have difficulty exercising on land [74]. Walking is similarly accessible and has been associated with a lower risk of cardiovascular events during follow-up [75]. Overall, exercise prescriptions should incorporate aerobic, resistance, and lower-impact modalities according to the individual’s clinical status, preferences, and functional capacity.

## Exercise Prescription

Exercise prescriptions should be individualized rather than standardized for all patients. The FITT framework (Frequency, Intensity, Time, and Type) provides a practical approach for translating general physical activity recommendations into individualized programs, including for adults with elevated blood pressure or cholesterol [76]. Moderate-intensity exercise performed daily or on most days of the week, with approximately 90–150 min or more of weekly activity depending on the clinical context, can contribute to blood-pressure control, while combining aerobic and resistance exercise may provide broader metabolic and lipid benefits [77–79]. Exercise frequency, intensity, duration, and modality should be adapted to cardiovascular risk, functional capacity, comorbidities, and individual goals. Functional capacity is particularly important when determining exercise intensity [80]. Before initiating an exercise program, clinicians should consider the patient’s medical history and relevant cardiovascular risk factors, particularly in individuals with hypertension-related target-organ damage, diabetes, frailty, cancer, or exercise intolerance. CAC scores and imaging-detected plaque can contribute to ASCVD risk assessment and decisions regarding preventive therapy; however, treatment decisions should incorporate shared decision-making rather than rely on CAC alone [81]. Accordingly, exercise prescriptions should consider CAC and other plaque characteristics together with functional capacity, symptoms, comorbidities, and the individual’s ability to safely adhere to the prescribed program.

### Exercise in Specific Patient Populations

Exercise prescriptions should account for differences in cardiovascular and metabolic risk across patient populations (Table 2). In older adults, exercise remains beneficial, but modality and intensity should be adapted to functional limitations, comorbidities, and baseline physical capacity. Comparative evidence suggests that different exercise modalities can produce distinct effects on endothelial function and arterial stiffness in older adults, supporting individualized rather than one-size-fits-all exercise prescriptions [82].

**Table 2:** Recommended exercise modalities according to population or clinical condition

Population	Recommended modality	Intensity / frequency	Key precautions	Refs.
Older adults	Combined/multicomponent exercise	Optimal intensity and frequency varied across individual randomized controlled trials; network meta-analysis findings support tailoring exercise prescriptions to individual vascular phenotypes.	Baseline assessment of functional capacity is recommended.	[82]
Type 2 diabetes	Combined aerobic + resistance training	Improves HbA1c regardless of exercise intensity or frequency according to the network meta-analysis.	Monitor the glycemic response, particularly when exercise is combined with glucose-lowering therapy.	[72]
Chronic kidney disease	Structured aerobic or combined exercise	Individualized according to renal function; exercise can reduce arterial stiffness, including PWV.	Exercise was historically restricted in this population but is generally safe when appropriately prescribed and monitored.	[83]
Postmenopausal women	Interval and combined training	Exercise prescription should be individualized to the population and functional capacity.	Accelerated vascular and atherosclerotic progression after menopause warrants attention to cardiovascular risk and vascular function.	[84]
Familial hypercholesterolemia	Exercise as an adjunct to pharmacotherapy	Exercise should not be considered a standalone intervention; it should complement intensive lipid-lowering therapy.	Exercise alone is insufficient to offset the substantial lifelong ASCVD risk associated with the genetic burden.	[85]
Cancer survivors	Cardio-oncology–integrated exercise	Individualized according to cardiotoxicity risk, cancer treatment history, and functional capacity.	Cardiovascular toxicity and vascular risk should be considered concurrently with exercise prescription.	[86]

For individuals with type 2 diabetes, combined aerobic and resistance training may be particularly useful. Such programs can improve arterial stiffness and HbA1c, addressing both vascular and metabolic risk factors [72]. Individuals with chronic kidney disease (CKD) may also benefit from exercise despite their elevated cardiovascular risk. Exercise has been shown to improve arterial stiffness in adults with CKD, although improvements in arterial stiffness do not necessarily translate into improvements in endothelial function [83].

Exercise is also important for postmenopausal women, in whom declining estrogen levels and ovarian function may adversely affect vascular health. Continuous endurance, interval, resistance, and hybrid training have all been associated with improvements in endothelial function, while interval training may provide particularly favorable effects on vascular health and arterial elasticity. In patients with familial hypercholesterolemia, exercise should complement (not replace) aggressive lipid-lowering therapy, particularly in individuals with severe lifelong LDL-C exposure and accelerated ASCVD risk. Homozygous familial hypercholesterolemia requires early diagnosis and intensive treatment [84–88].

Physical activity may also mitigate treatment-related cardiovascular risks in cancer survivors. In oncology populations, exercise interventions can safely improve cardiorespiratory fitness, muscular strength, and quality of life while helping preserve cardiovascular function during potentially cardiotoxic therapies [86].

## Safety Considerations

Although exercise provides substantial cardiovascular benefits, safety considerations are particularly relevant for individuals with coronary plaque, high CAC scores, established ischemic heart disease, or plans for vigorous exercise [89]. Long-term, high-volume endurance exercise may be associated with greater coronary atherosclerotic burden in some athletes, although the presence of coronary plaque does not automatically make exercise unsafe [90]. Extreme endurance athletes may have a higher prevalence of coronary atherosclerosis without necessarily having a greater prevalence of high-risk plaque [91]. Individuals with substantial plaque burden or cardiovascular symptoms should therefore undergo appropriate cardiovascular evaluation before initiating vigorous exercise. Rather than routinely restricting physical activity, clinical decisions should incorporate symptoms, functional capacity, plaque characteristics, disease severity, and the physiological demands of the planned activity [92–94]. This approach preserves exercise as a central component of cardiovascular prevention while prioritizing individualized safety.

## Translational Application of Exercise

### Exercise and Medical Therapy

Structured exercise may contribute to slowing or modifying atherosclerotic disease, and its combination with medical therapy may provide complementary benefits. Exercise has demonstrated favorable effects on vascular and metabolic health and therefore represents an important nonpharmacological component of ASCVD management [95, 96]. When combined with statin therapy, moderate-intensity exercise has not been shown to worsen statin-associated muscle symptoms and may improve muscle performance, mitochondrial function, and quality of life [96]. Similarly, a placebo-controlled trial demonstrated that exercise combined with the glucagon-like peptide-1 receptor agonist (GLP-1 RA) liraglutide produced greater improvements in cIMT and inflammatory markers than either intervention alone, with exercise appearing to contribute substantially to the vascular and anti-inflammatory effects [97]. The main clinical trials investigating the effects of exercise on subclinical atherosclerosis are summarized in Table 3. Other clinical evidence suggests that, although pharmacological treatment with agents such as rosuvastatin and olmesartan can improve blood pressure, lipid profiles, and glucose levels, exercise may provide additional effects on vascular pathophysiology [98]. The interaction between exercise and other pharmacological therapies, including PCSK9 inhibitors, warrants further investigation because physical activity may influence pathways involved in PCSK9 regulation [99]. Nonpharmacological interventions may also be complementary: exercise combined with a Mediterranean-style diet may produce more favorable metabolic effects than either strategy considered in isolation [100, 101].

**Table 3:** Characteristics of main randomized clinical trials on exercise and subclinical atherosclerosis-related endpoints

Study (First Author, Year) [Ref.]	Population	Intervention	Comparator	Duration	Primary Outcome(s)	Key Findings
Sandsdal et al., 2026 (S-LiTE) [97]	130 adults with obesity	Exercise ± liraglutide (4 groups: exercise, liraglutide, combination, placebo)	Liraglutide alone; placebo	52 weeks	cIMT, inflammatory cytokines, endothelial biomarkers	Exercise alone or combined with liraglutide reduced cIMT and IL-6/IFN-γ; combination also improved sICAM-1, sVCAM-1, tPA; liraglutide alone showed no vascular improvement
Ghardashi Afousi et al., 2026 [63]	60 adults with hypertension (age ~ 60)	HIIT (cycling, 1 min at 85–90% HRR, 3×/week)	Usual care control	12 weeks	Ambulatory BP, cfPWV, gut-derived metabolites	HIIT improved ambulatory BP, arterial stiffness (cfPWV), and gut metabolites (TMAO, SCFAs)
Yan et al., 2026 [67]	56 patients with essential hypertension	HIIT (1 × 10 min with 2-min intervals, 3×/week)	MICT (35 min, 3×/week)	8 weeks	FMD (primary); BP; cardiovascular function	Both HIIT and MICT improved FMD; HIIT showed additional benefits on inflammation and oxidative stress
Fatehi et al., 2026 [73]	45 men with type 2 diabetes	Low-load BFR resistance training (20–30% 1RM)	High-resistance training (70–80% 1RM); control	16 weeks	SIRT1, NO, ET-1, BP	BFR resistance training improved SIRT1 and NO; both training groups improved endothelial markers vs. control
Choi et al., 2026 [102]	28 patients with hypertension (age ~ 61)	Inspiratory muscle strength training (30 breaths at 75% MIP, 5×/week)	Aerobic exercise training (70% HRR, 30 min, 5×/week)	8 weeks	FMD, cfPWV, augmentation index	Both IMST and AET improved vascular function; comparative effectiveness assessed
Hernández-López et al., 2026 [103]	22 sedentary women	Exercise snacks (12-min daily sessions)	Physically active comparison group	8 weeks	Subclinical atherosclerosis markers, mitochondrial dynamics, body composition	ExS improved metabolic markers, body composition, PBMC mitochondrial dynamics, and subclinical atherosclerosis markers
Yildirim Ayaz et al., 2026 [104]	64 adults with prediabetes (age ~ 47)	Supervised moderate-intensity aerobic exercise (50–70% HRmax, 3×/week)	Standard lifestyle advice	12 weeks	miR-126 expression, cIMT, ABI	Exercise increased miR-126 expression and improved cIMT and ABI
Barbosa et al., 2024 [100]	Adults ≥ 50 years with high CVD risk (SCORE2)	Mediterranean diet + physical exercise (3 groups: intervention, partial intervention, no intervention)	No intervention	12 weeks	Anthropometric and biochemical CVD risk factors	Mediterranean diet + exercise improved anthropometric and biochemical CVD risk factors
Allard et al., 2021 [79]	Symptomatic and asymptomatic statin users	Moderate-intensity exercise training	Sedentary statin users	12 weeks	Skeletal muscle performance, mitochondrial function, fiber capillarization	Exercise improved muscle performance and mitochondrial function without worsening statin-associated muscle symptoms

## Biomarkers

Several biomarkers may help evaluate the effects of exercise interventions on atherosclerotic risk. The 20-year ATTICA prospective study identified apolipoprotein B (apoB) as an important marker, with elevated apoB levels associated with higher ASCVD incidence independently of LDL-C, non-HDL cholesterol, and lipoprotein(a) [105]. High-sensitivity C-reactive protein (hs-CRP) and peak oxygen consumption (VO₂ peak) may provide additional information [106, 107]. Exercise interventions can reduce hs-CRP, reflecting attenuation of systemic inflammation [59], while impaired vascular and cardiovascular function may be associated with lower VO₂ peak [108, 109]. Accordingly, apoB, hs-CRP, and VO₂ peak may provide complementary measures for evaluating the response to exercise interventions. Wearable-device and telemonitoring approaches may further facilitate individualized exercise prescription and monitoring of physical activity and cardiorespiratory fitness [110].

## Clinical Guidelines and Integration of Emerging Technologies

### Emerging Technologies

Emerging technologies are increasingly supporting personalized cardiovascular prevention. Artificial intelligence (AI) can quantify coronary calcium with accuracy comparable to expert assessment while substantially reducing analysis time, using deep-learning approaches such as convolutional neural networks [108, 111, 112]. Digital-twin approaches combined with coronary CT angiography and fractional flow reserve (CT-FFR) have also been investigated for predicting atherosclerotic disease progression [113]. In addition, a tactile-transparent wearable (TTW) sensor system combined with Doppler ultrasound has been used to assess carotid-radial PWV and demonstrated associations between arterial stiffness, age, and body mass index [114]. More broadly, machine-learning approaches can integrate clinical variables beyond traditional risk factors and may improve individualized cardiovascular risk stratification [115].

### Current Guidelines

Current cardiovascular guidelines increasingly emphasize individualized risk assessment and treatment. The 2025 ESC/EAS focused update recommends considering CAC and imaging-detected subclinical atherosclerosis as risk modifiers in individuals at moderate risk or near treatment thresholds [116]. The 2026 ACC/AHA guideline recommends measuring lipoprotein(a) at least once and using the PREVENT-ASCVD equations for risk estimation [117]. For athletes, cardiovascular evaluation is recommended when abnormalities or relevant risk factors are present, particularly before participation in high-intensity exercise [118, 119]. Overall, these recommendations support individualized clinical decision-making rather than broad restrictions on exercise or treatment.

## Information Necessary to Modify Existing Clinical Practice

Prior reviews have examined the general cardiovascular benefits of exercise or the role of physical activity in primary prevention. This review adds to the existing literature in several ways. It integrates the mechanistic pathways by which exercise modifies atherosclerotic vascular disease (including shear stress-mediated endothelial nitric oxide synthase activation, myokine signaling, macrophage phenotype modulation, endothelial progenitor cell mobilization, and plaque composition changes) with the clinical evidence demonstrating measurable improvements in vascular function and structure. By framing exercise specifically as a potential disease-modifying intervention for individuals with imaging-detected subclinical atherosclerosis, rather than as a generic preventive measure, this review provides a mechanistically grounded rationale for exercise prescription in this population. The review also addresses the clinically important “calcium paradox” by offering a mechanistic interpretation that distinguishes exercise-associated dense calcification from high-risk lipid-rich plaque accumulation. Furthermore, it synthesizes modality-specific and population-specific evidence into practical recommendations, identifies circulating biomarkers and imaging tools that may guide individualized exercise prescriptions, and highlights the complementary role of exercise alongside pharmacological therapies, including statins and glucagon-like peptide-1 receptor agonists.

Several findings synthesized in this review carry implications that extend beyond current standard practice. Meta-analytic evidence demonstrates that exercise training improves endothelial function (flow-mediated dilation), reduces arterial stiffness (pulse wave velocity), and decreases carotid intima-media thickness, supporting the concept that exercise functions as a disease-modifying intervention in subclinical atherosclerosis rather than solely as a primary-prevention strategy [54, 55, 57].

These vascular improvements are observed across diverse populations, including older adults, patients with type 2 diabetes, and postmenopausal women. Modality-specific effects have emerged with sufficient consistency to inform prescription. Interval training shows favorable effects on arterial stiffness, while combined aerobic-resistance training appears to maximize improvements in endothelial function [55, 68, 69]. This evidence supports a shift from generic physical activity advice toward individualized, modality-specific exercise prescriptions in patients with imaging-detected subclinical atherosclerosis. The reinterpretation of the exercise-associated coronary artery calcium increase has clinical relevance. Rather than indicating worsening disease, exercise-related coronary artery calcium increases in highly active individuals may reflect a shift toward densely calcified, potentially more stable plaque, as opposed to the lipid-rich, vulnerable plaques more commonly observed in sedentary individuals. While this interpretation does not diminish the prognostic value of coronary artery calcium in risk stratification, it suggests that exercise-associated calcification should not automatically be equated with disease progression, and it supports continued exercise participation in appropriately evaluated individuals.

The S-LiTE randomized trial demonstrated that exercise, alone or combined with the glucagon-like peptide-1 receptor agonist liraglutide, reduced carotid intima-media thickness and systemic pro-inflammatory cytokines during weight-loss maintenance, whereas liraglutide alone did not [97]. This finding suggests that exercise provides vascular and anti-inflammatory benefits that pharmacotherapy alone may not achieve, supporting the integration of structured exercise into comprehensive risk-management plans for patients receiving glucagon-like peptide-1 receptor agonist therapy.

Finally, current guidelines increasingly emphasize individualized risk assessment incorporating coronary artery calcium, imaging-detected plaque, and novel biomarkers [81, 116, 117]. The evidence reviewed here supports using these tools not only for risk stratification but also for guiding individualized exercise prescriptions, including decisions about exercise intensity, modality, and safety evaluation in patients with established subclinical atherosclerosis.

Several limitations of this review should be acknowledged. We did not employ a systematic search strategy, formal risk-of-bias assessment, or grading of evidence quality; the selection and emphasis of studies reflect the authors’ judgment of clinical relevance, introducing the potential for selection bias. Substantial heterogeneity exists across the included studies with respect to exercise modalities, intensities, durations, participant populations, and methods of measuring vascular endpoints, which limits direct comparability and the strength of pooled conclusions [102, 103]. The majority of clinical evidence relies on surrogate endpoints (carotid intima-media thickness, pulse wave velocity, flow-mediated dilation, and coronary artery calcium scores) rather than hard cardiovascular outcomes such as myocardial infarction, stroke, or cardiovascular mortality. While these surrogate markers are validated predictors of cardiovascular risk, their improvement does not necessarily confirm a reduction in clinical events.

## Knowledge Gaps

Despite the growing body of evidence, several knowledge gaps persist. The optimal exercise dose (encompassing frequency, intensity, duration, and modality) for different subclinical atherosclerosis phenotypes (e.g. noncalcified plaque, high coronary artery calcium scores, elevated arterial stiffness) has not been established. It remains unknown whether exercise-induced improvements in surrogate vascular markers translate into a reduction in hard cardiovascular events in populations with confirmed subclinical atherosclerosis, as no dedicated randomized trial has been powered to detect such outcomes. The long-term effects of exercise on plaque composition, including the balance between dense calcification and lipid-rich vulnerable plaque, and their relationship to clinical outcomes require clarification. The interaction between exercise and newer pharmacotherapies remains largely unexplored in the context of subclinical atherosclerosis. Sex-specific, age-specific, and ethnicity-specific exercise responses in subclinical atherosclerosis populations have not been adequately characterized, despite known differences in plaque burden and calcification patterns across demographic groups. The role of circulating biomarkers, including myokines, apolipoprotein B, and high-sensitivity C-reactive protein, in guiding and monitoring exercise prescriptions has not been validated in prospective trials. Finally, whether the exercise-associated coronary artery calcium increase represents true plaque stabilization with clinical benefit or merely accelerated calcification without prognostic improvement remains an unresolved question of direct clinical importance.

## Future Directions

Future research should prioritize several areas to address the identified knowledge gaps. Adequately powered randomized controlled trials with imaging endpoints (particularly serial coronary computed tomography angiography for plaque characterization and coronary artery calcium progression) are needed in populations with imaging-confirmed subclinical atherosclerosis to determine whether exercise can modify plaque phenotype and reduce cardiovascular events. Biomarker-guided exercise prescription trials, integrating apolipoprotein B, high-sensitivity C-reactive protein, peak oxygen consumption, and emerging myokine panels, may enable individualized exercise recommendations tailored to vascular phenotype and treatment response. Mechanistic studies should explore recently identified pathways, including exercise-induced extracellular vesicle signaling, the modulation of clonal hematopoiesis of indeterminate potential [120], and the gut microbiome-vascular axis, as potential mediators of the atheroprotective effects of exercise. Longitudinal studies extending beyond two years are needed to assess the durability of exercise-induced vascular improvements and their impact on plaque regression and hard cardiovascular outcomes. The integration of artificial intelligence and digital health technologies (including machine learning–based risk stratification, wearable sensors for continuous vascular monitoring, and telemonitoring for exercise adherence) offers promising avenues for precision exercise medicine. Finally, precision exercise approaches that tailor modality and intensity to plaque phenotype, genetic risk profile, and comorbidity burden represent a key direction for translating the evidence reviewed here into individualized clinical practice.

## Conclusions

The evidence reviewed in this article supports several conclusions. Exercise training improves endothelial function, as measured by flow-mediated dilation, reduces arterial stiffness, as measured by pulse wave velocity, and decreases carotid intima-media thickness, with these effects supported by meta-analytic evidence across multiple populations. Modality-specific effects have been demonstrated: interval training shows favorable effects on arterial stiffness, while combined aerobic-resistance training appears to maximize improvements in endothelial function. The exercise-associated increase in coronary artery calcium observed in highly active individuals may reflect a shift toward densely calcified, potentially more stable plaque rather than accumulation of high-risk lipid-rich lesions, although the clinical significance of this finding remains uncertain and requires further investigation. On the basis of this evidence, exercise should be integrated into cardiovascular risk management for individuals with subclinical atherosclerosis as an individualized, disease-modifying adjunct to conventional risk-factor modification, with prescriptions tailored to atherosclerotic burden, plaque phenotype, functional capacity, and comorbidities. However, the available evidence is derived predominantly from studies using surrogate vascular endpoints, and whether exercise directly reduces hard cardiovascular events in populations with confirmed subclinical atherosclerosis awaits dedicated randomized trials.

## Key References

- Hsu JJ, Tintut Y, Demer LL. The paradox of exercise and coronary artery calcification: potential underlying mechanisms. Circ Res. 2025;137(2):335–49. https://doi.org/10.1161/CIRCRESAHA.125.326011.
  - ○ A perspective synthesis that examines the potential of underlying cellular and molecular mechanisms of the athlete calcification paradox.
- Omole JG, Okon IA, Udom GJ, Aziakpono OM, Agbana RD, Aturamu A, et al. Neurophysiological mechanisms underlying cardiovascular adaptations to exercise: A narrative review. Physiol Rep. 2025;13(13):e70439. https://doi.org/10.14814/phy2.70439.
  - ○ This paper examines neurophysiological and molecular pathways regulating cardiovascular and vascular adaptations to exercise.
- Li Q, Mao W, Lu Y, Lu T, Xu X, Pan Y, et al. Exercise improves atherosclerotic plaque stability through macrophage autophagy and the FGF21 signaling pathway. Int J Mol Sci. 2026;27(11):4996. https://doi.org/10.3390/ijms27114996.
  - ○ An experimental preclinical study in ApoE-deficient mice proving the effects of aerobic exercise training on atherosclerotic plaque stability, macrophage autophagy, and FGF21 signaling pathway.
- Barbosa AR, Pais S, Marreiros A, Correia M. Impact of a Mediterranean-Inspired Diet on Cardiovascular Disease Risk Factors: A Randomized Clinical Trial. Nutrients. 2024;16(15):2443. https://doi.org/10.3390/nu16152443.
  - ○ This randomized controlled trial evaluated the impact of a Mediterranean-inspired diet combined with physical exercise on cardiovascular disease risk factors in adults aged 50 and older with high cardiovascular risk and no history of acute myocardial infarction.
- Suárez-Cuenca JA, Montoya-Ramírez J, Gutiérrez-Buendía JA, Vera-Gómez E, Hernández-Patricio A, Vargas-Cuautle ME, et al. Pulse-wave velocity, flow-mediated dilation, and carotid intima-media thickness to assess cardiovascular risk in population with metabolic syndrome. J Vis Exp. 2024;(211):e65842. https://doi.org/10.3791/65842.
  - ○ A translational research protocol that examines techniques for measuring arterial stiffness, endothelial dysfunction, and atherogenesis through pulse-wave velocity, flow-mediated dilation, and carotid intima-media thickness to evaluate cardiovascular risk in populations with metabolic syndrome.
- Thangaraj PM, Benson SH, Oikonomou EK, Asselbergs FW, Khera R. Cardiovascular care with digital twin technology in the era of generative artificial intelligence. Eur Heart J. 2024;45(45):4808-4821. https://doi.org/10.1093/eurheartj/ehae619.
  - ○ This review examines digital twin technology and generative artificial intelligence for patients with cardiovascular disease to summarize current clinical applications, technical advances, and key implementation challenges in precision cardiology.

## Data Availability

No datasets were generated or analysed during the current study.

## Abbreviations

ACC: American College of Cardiology
AI: Artificial Intelligence
AHA: American Heart Association
apoB: Apolipoprotein B
ASCVD: Atherosclerotic Cardiovascular Disease
BDNF: Brain-Derived Neurotrophic Factor
CAC: Coronary Artery Calcium
CCTA: Coronary Computed Tomography Angiography
CD36: Cluster of Differentiation 36
cIMT: Carotid Intima-Media Thickness
CKD: Chronic Kidney Disease
CT: Computed Tomography
CT-FFR: Computed Tomography–Derived Fractional Flow Reserve
EAS: European Atherosclerosis Society
eNOS: Endothelial Nitric Oxide Synthase
EPC: Endothelial Progenitor Cell
ESC: European Society of Cardiology
FITT: Frequency, Intensity, Time, and Type
FGF21: Fibroblast Growth Factor 21
FMD: Flow-Mediated Dilation
GLP-1 RA: Glucagon-Like Peptide-1 Receptor Agonist
HDL: High-Density Lipoprotein
HIIT: High-Intensity Interval Training
ICAM-1: Intercellular Adhesion Molecule-1
IL-6: Interleukin-6
LDL: Low-Density Lipoprotein
LDL-C: Low-Density Lipoprotein Cholesterol
LOX-1: Lectin-Like Oxidized LDL Receptor-1
LSS: Laminar Shear Stress
MCP-1: Monocyte Chemoattractant Protein-1
Metrnl: Meteorin-Like Protein
MMPs: Matrix Metalloproteinases
MSTN: Myostatin
NF-κB: Nuclear Factor Kappa B
NLRP3: NLR Family Pyrin Domain Containing 3
NO: Nitric Oxide
PCSK9: Proprotein Convertase Subtilisin/Kexin Type 9
PWV: Pulse Wave Velocity
ROS: Reactive Oxygen Species
TNF-α: Tumor Necrosis Factor-Alpha
TTW: Tactile-Transparent Wearable
VCAM-1: Vascular Cell Adhesion Molecule-1
VEGF: Vascular Endothelial Growth Factor
VO₂ peak: Peak Oxygen Consumption
